# Supplementary material for: Contributing factors to the oxidation-induced mutational landscape in human cells
Source: Nat Commun. 2024 Dec 23;15:10722. doi: 10.1038/s41467-024-55497-z (PMC11666792; doi:10.1038/s41467-024-55497-z)
Supplement: Supplementary file 1 — Supplementary Information [file 41467_2024_55497_MOESM1_ESM.pdf]

**Supplementary information:**

**Supplementary Data 1: List of mutations from whole genome sequencing of WT, HMCES-deficient, and Pol η-deficient RPE-1 cells.** Related to Figures 1-3 and 5-6.

**Supplementary Table 1: Cryo-EM data collection and processing statistics.** Related to Figure 4.

| **Data collection and processing** | | | | |
|---|---|---|---|---|
| **Dataset** | **OGG1-8oxoG-NCP$^{-6}$** | | **OGG1-8oxoG-NCP$^{+4}$** | |
| Magnification | 81,000x | | 81,000x | |
| Voltage (kV) | 300 | | 300 | |
| Electron exposure ($e^{-}$/Å$^{2}$) | 60 | | 60 | |
| Defocus range (μm) | −0.5 to −2.5 | | −0.5 to −2.5 | |
| Pixel size (Å) | 0.534 | | 0.534 | |
| Symmetry imposed | C1 | | C1 | |
| Initial particle number | 4,166,129 | | 3,850,706 | |
| **Structure** | **8oxoG-NCP$^{-6}$** | **OGG1-8oxoG-NCP$^{-6}$** | **8oxoG-NCP$^{+4}$** | **OGG1-8oxoG-NCP$^{+4}$** |
| Final particle number | 44,791 | 32,685 | 57,304 | 19,114 |
| Map resolution (Å) | 3.1 | 3.3 | 3.0 | 3.6 |
| FSC threshold | 0.143 | 0.143 | 0.143 | 0.143 |
| PDB accession | 8VWS | 8VWT | 8VWU | 8VWV |
| EMDB accession | EMD-43595 | EMD-43596 | EMD-43600 | EMD-43601 |
| **Refinement** | | | | |
| Initial model used (PDB ID) | 7U52 | 7U52, 1EBM | 7U52 | 7U52, 1EBM |
| Model resolution (Å) | 3.2 | 3.6 | 3.1 | 3.9 |
| FSC threshold | 0.5 | 0.5 | 0.5 | 0.5 |
| **Model composition** | | | | |
| Nonhydrogen atoms | 11,907 | 14,451 | 12021 | 14,474 |
| Protein residues | 743 | 1,061 | 757 | 1,066 |
| Nucleotide | 294 | 294 | 294 | 294 |
| **B factors (Å$^{2}$ )** | | | | |
| Protein | 31.22 | 83.65 | 22.52 | 146.82 |
| Nucleotide | 80.91 | 111.48 | 61.90 | 184.59 |
| **r.m.s. deviations** | | | | |
| Bond Length (Å) (# > 4σ) | 0.004 (0) | 0.005 (2) | 0.003 (0) | 0.005 (0) |
| Bond Angles ($^{o}$) (# > 4σ) | 0.619 (1) | 0.886 (33) | 0.558 (0) | 0.868 (27) |
| **Validation** | | | | |
| MolProbity score | 1.27 | 1.67 | 1.23 | 1.53 |
| Clashscore | 5.10 | 6.92 | 4.53 | 6.15 |
| Poor rotamers (%) | 0.00 | 0.79 | 0.00 | 0.68 |
| **Ramachandran plot** | | | | |
| Favored (%) | 98.21 | 95.87 | 98.65 | 96.85 |
| Allowed (%) | 1.79 | 4.13 | 1.35 | 3.15 |
| Disallowed (%) | 0.00 | 0.00 | 0.00 | 0.00 |

**Supplementary Table 2: Oligonucleotides used for cryo-EM structures of OGG1 bound to 8-oxoG lesions in nucleosomes.** Related to Figure 4.

| Oligo | Sequence (5′ – 3′) |
|---|---|
| **8oxoG1-NCP oligos** | |
| 8oxoG-NCP−6 (I-strand) | ATCGAGAATCCCGGTGCCGAGGCCGCTCAATTGGTCGTAGACAGCTCTAGCACC<br>GCTTAAACGCACGTACGCGCTGTCCCCCGCGTTTTAACCGCCAAGGGGATTACT<br>CCC TAGTCT CCAGGCACGTGTCAGATCTATACATCCGAT |
| 8oxoG-NCP−6 (J-strand) | ATCGGATGTATA**[8oxoG]**ATCTGACACGTGCCTGGAGACTAGGGAGTAATCCCCTT<br>GGCGGTTAAAACGCGGGGGACAGCGCGTACGTGCGTTTAAGCGGTGCTAGAGCT<br>GTCTACGACCAATTGAGCGGCCTCGGCACCGGGATTCTCGAT |
| **8oxoG2-NCP oligos** | |
| 8oxoG-NCP+4 (I-strand) | ATCGAGAATCCCGGTGCCGAGGCCGCTCAATTG**[8oxoG]**TCGTAGACAGCTCTAGC<br>ACCGCTTAAACGCACGTACGCGCTGTCCCCCGCGTTTTAACCGCCAAGGGGATTA<br>CTCCCTAGTCTCCAGGCACGTGTCAGATATATACATCCGAT |
| 8oxoG-NCP+4 (J-strand) | ATCGGATGTATATATCTGACACGTGCCTGGAGACTAGGGAGTAATCCCCTTGGCG<br>GTTAAAACGCGGGGGACAGCGCGTACGTGCGTTTAAGCGGTGCTAGAGCTGTCT<br>ACGACCAATTGAGCGGCCTCGGCACCGGGATTCTCGAT |

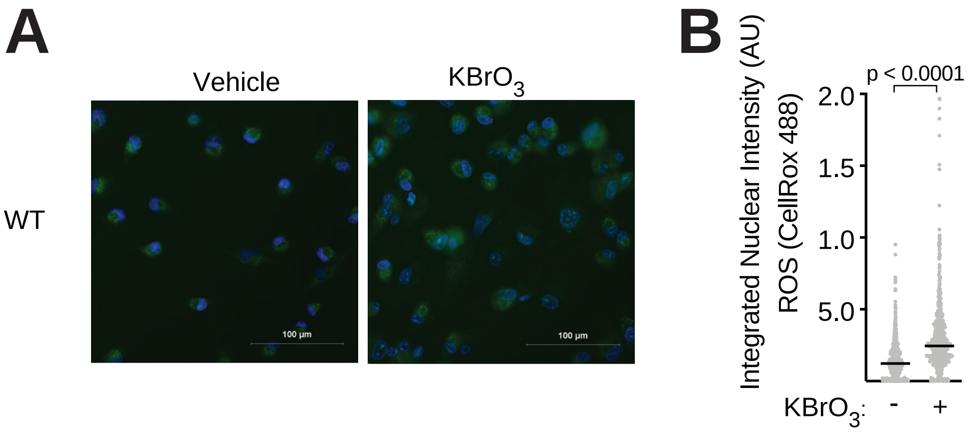


**Supplementary Figure 1: Modest induction of Cellular ROS by Potassium Bromate Treatment. A**, Fluorescent ROS signal localization was captured by fluorescent imaging. WT RPE-1 cells were treated with 250 µM $KBrO_3$ and CellROX 488 ROS reagent (green) for thirty minutes. DNA was subsequently stained with Hoechst 33342 (blue), and live cells were imaged. **B**, Quantitation of the integrated nuclear ROS intensity. Circles indicate independent cells quantified and horizontal bars represent median intensities. p-value < 0.0001 were derived from an ANOVA with Dunn's multiple comparisons.

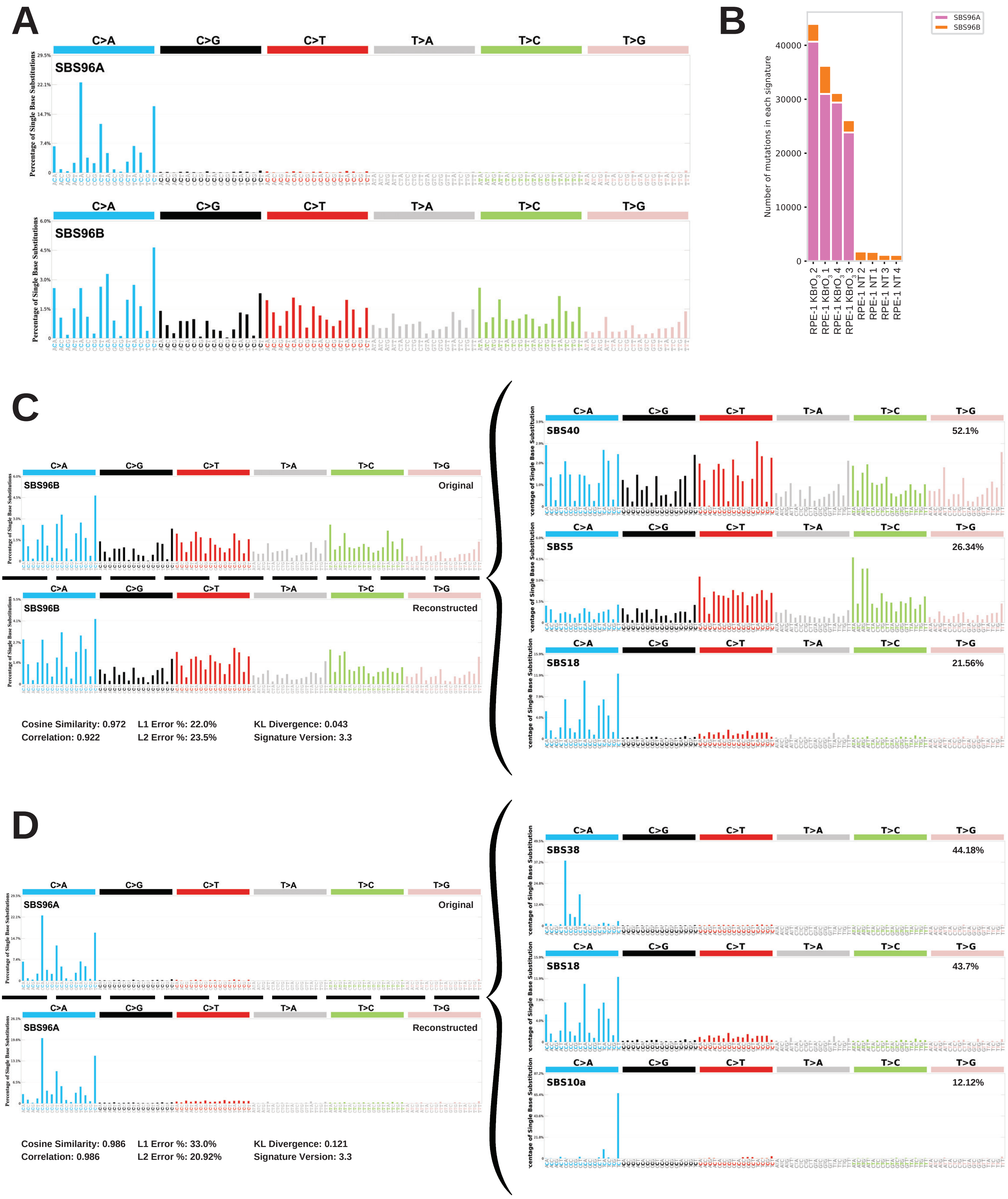


**Supplementary Figure 2: Non-negative matrix factorization (NMF) mutation signature generation from $KBrO_3$-treated and untreated cells.**

**A,** Determination of de novo mutation signatures in $KBrO_3$-treated and untreated WT RPE-1 cells by NMF resulted in **B**, the activity of SBS96A was present only in the treated cells indicating it results from $KBrO_3$ exposure and that SBS96B is background mutagenesis. **C**, The COSMIC deconvolution of SBS96B (the untreated cell culture signature) and percentage attribution. **D**, The COSMIC deconvolution of SBS96A (the $KBrO_3$-induced cell culture signature) and percentage attribution led to a cosine similarity of 0.986. Related to Figure 1.

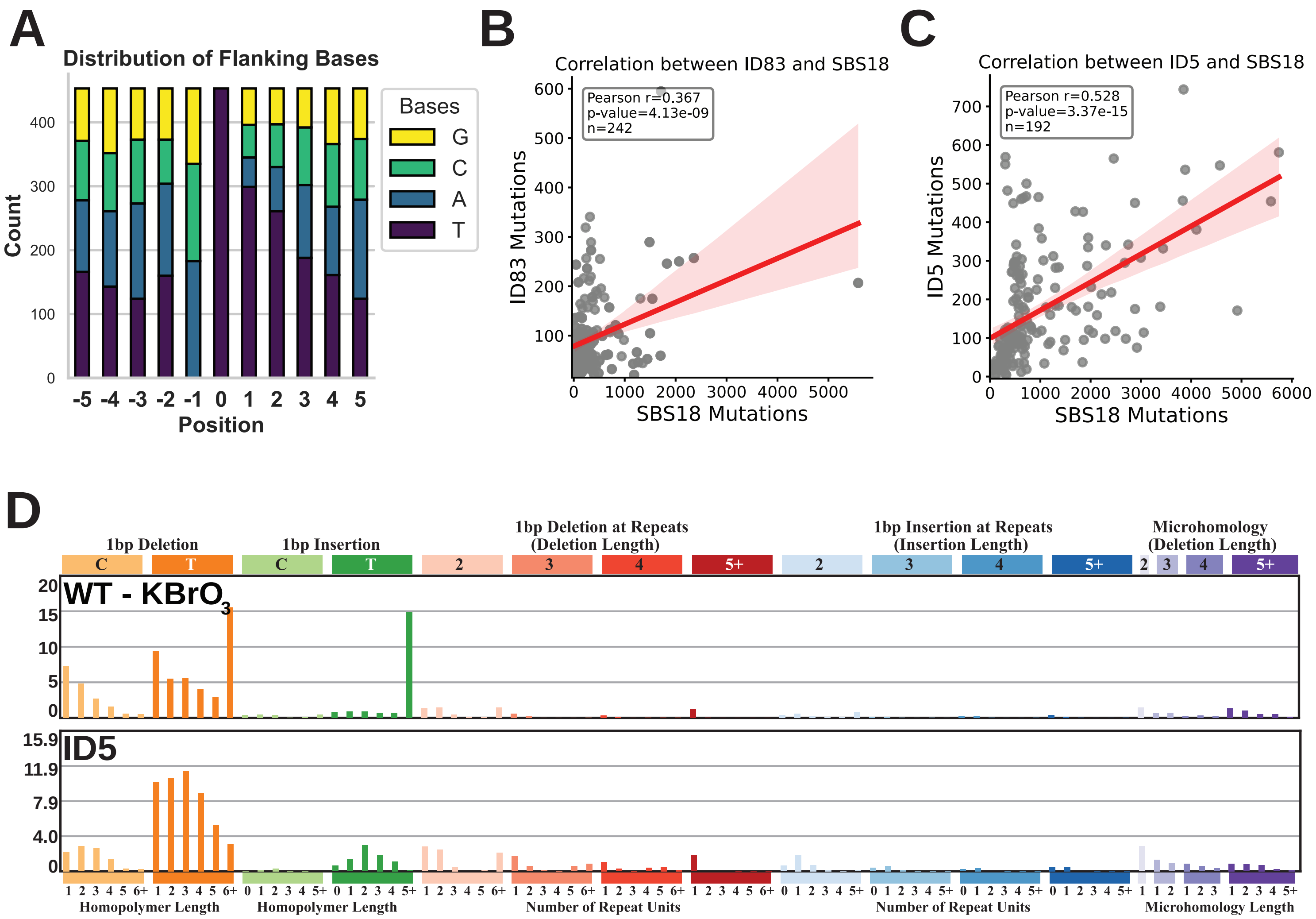


**Supplementary Figure 3: Correlation of $KBrO_3$-induced INDEL signature with COSMIC SBS Signatures.**

**A,** Fractional breakdown of each nucleotide in positions relative to a single deleted T base. The overrepresentation of T on the right and not left is due to the convention of mutation callers left aligning the deleted base in homopolymers. **B**, Correlation the $KBrO_3$-induced of INDEL signature (ID83) with SBS18 in primary tumor samples. Red line indicates a linear trendline, the transparent red shaded area represents the 95% confidence interval, and individual tumors are represented by circles. **C**, Correlation of COSMIC ID5 with SBS18. Red line, red shaded area, and circles are as in B. **D**, Similarity of $KBrO_3$-induced ID83 and COSMIC ID5 (Cosine similarity = 0.6116). Related to Figure 1.

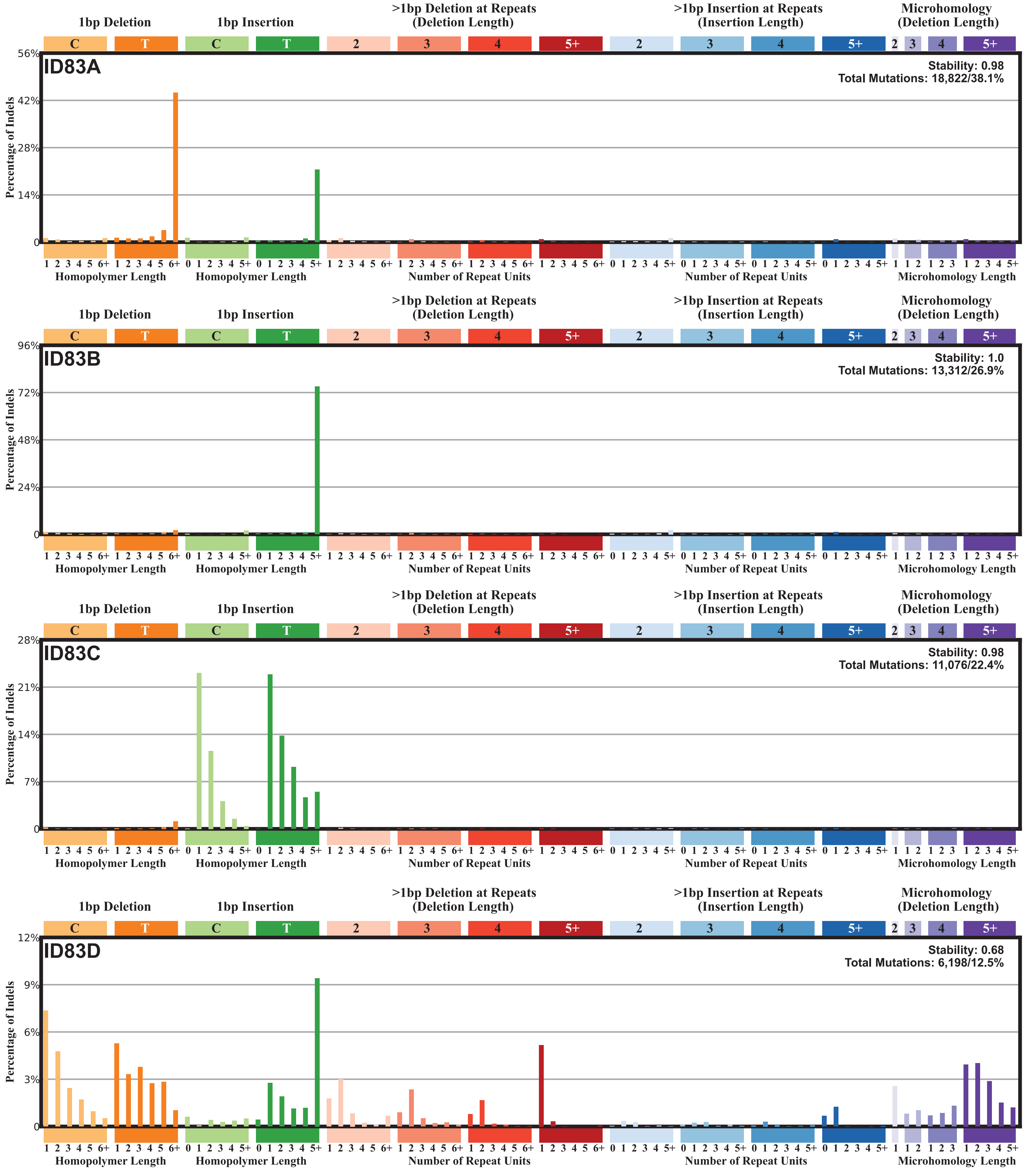


**Supplementary Figure 4: NMF Analysis on PCAWG tumors filtered for a minimum of 25% SBS18 mutation contribution in their spectra.**
The mutation signature output from the INDEL NMF results with associated percentage makeup and mutation count of the INDEL spectra. Related to Figure 1.

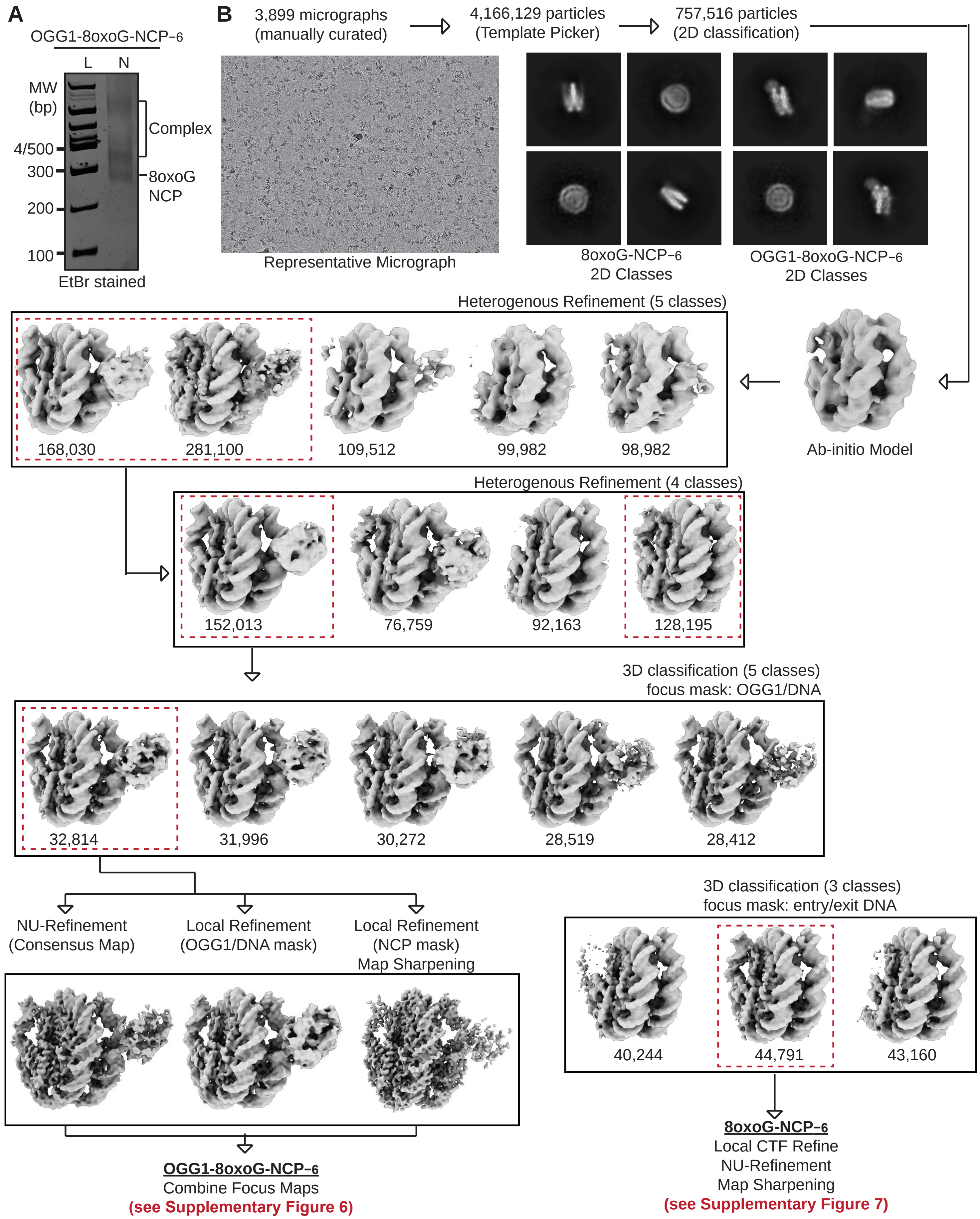


**Supplementary Figure 5: Single particle analysis of OGG1-8-oxoG-NCP$_{-6}$.**
**A,** Native PAGE gel of the OGG1-8-oxoG-NCP$_{-6}$ cryo-EM sample (S) and a 100 bp DNA ladder (L). The nucleosome complex was visualized with ethidium bromide staining. **B**, Flowchart of the data processing pipeline for the OGG1-8-oxoG-NCP$_{-6}$ cryo-EM dataset. A representative micrograph (n=3,899) and representative 2D classes from the OGG1-8-oxoG-NCP$_{-6}$ cryo-EM dataset are shown. All maps chosen for the downstream processing are labeled by a dotted red box. The final maps, models, and quality assessment for the OGG1-8-oxoG-NCP$_{-6}$ and 8-oxoG-NCP$_{-6}$ structures can be found in Supplementary Figure 6 and 7, respectively. Related to Figure 4.

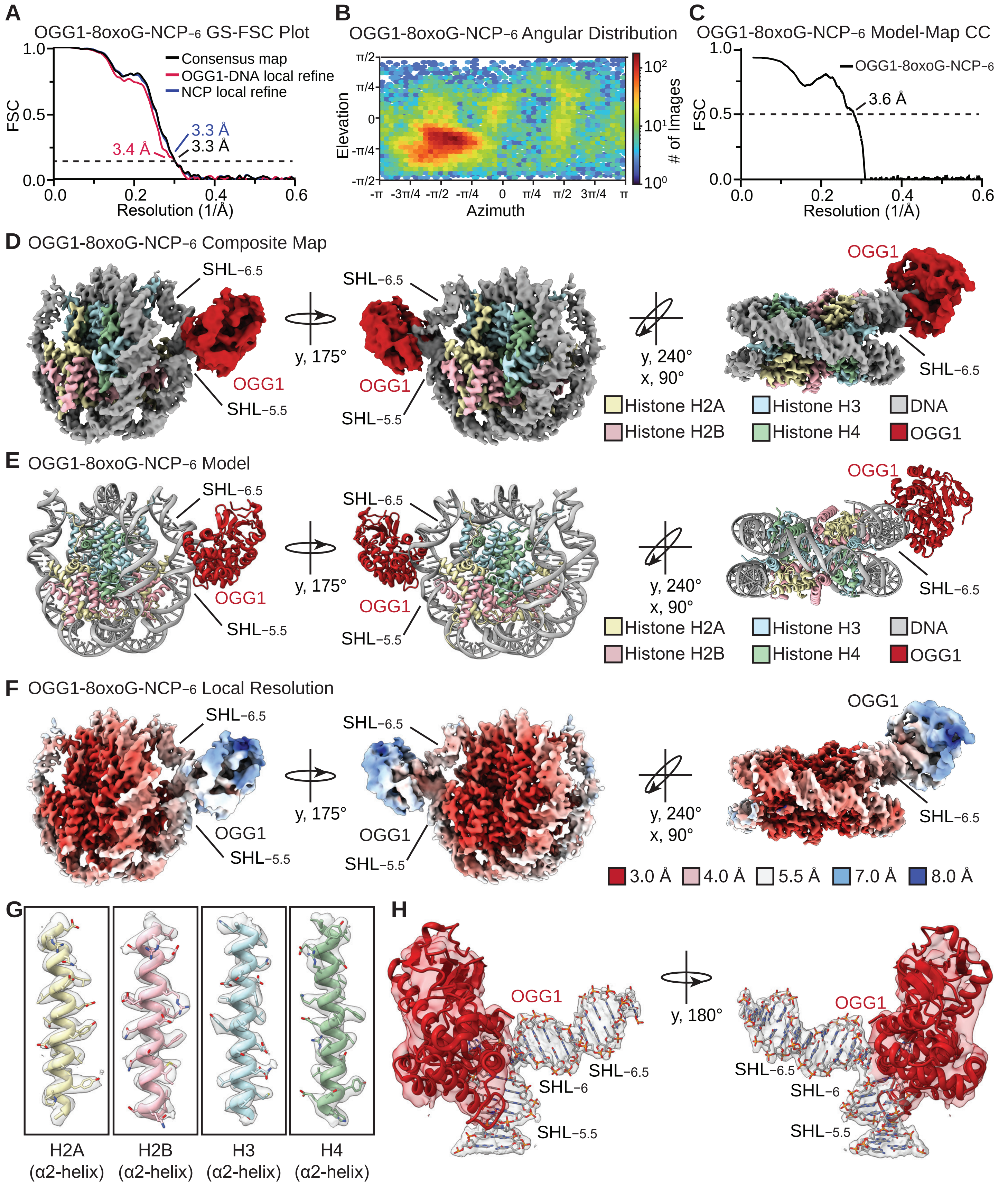


**Supplementary Figure 6: OGG1-8-oxoG-NCP$_{-6}$ map and model quality assessment.**
**A,** Gold-standard Fourier shell correlation (GS-FSC) curves for the OGG1-8-oxoG-NCP$_{-6}$ (consensus, black line), OGG1-8-oxoG-NCP$_{-6}$ (NCP focus, blue line), and OGG1-8-oxoG-NCP$_{-6}$ (OGG1/DNA focus, red line) cryo-EM maps. The dashed line corresponds to FSC-0.143. **B**, Angular distribution heatmap for the 8-oxoG-NCP$_{-6}$ cryo-EM maps. **C**, Map-to-model FSC curves for the OGG1-8-oxoG-NCP$_{-6}$ model and OGG1-8-oxoG-NCP$_{-6}$ composite cryo-EM map. **D**, Final 3.3 Å OGG1-8-oxoG-NCP$_{-6}$ composite cryo-EM map shown in three different orientations. **E**, Final OGG1-8-oxoG-NCP$_{-6}$ model shown in three different orientations. **F**, Local resolution estimation for the OGG1-8-oxoG-NCP$_{-6}$ composite cryo-EM map shown in three different orientations. **G**, Representative segmented density for histones H2A, H2B, H3, and H4 in the OGG1-8-oxoG-NCP$_{-6}$ composite cryo-EM map. **H**, Representative segmented density for OGG1 and the nucleosomal DNA (SHL$_{-5.5}$ to SHL$_{-6.5}$) in the OGG1-8-oxoG-NCP$_{-6}$ cryo-EM map. Related to Figure 4.

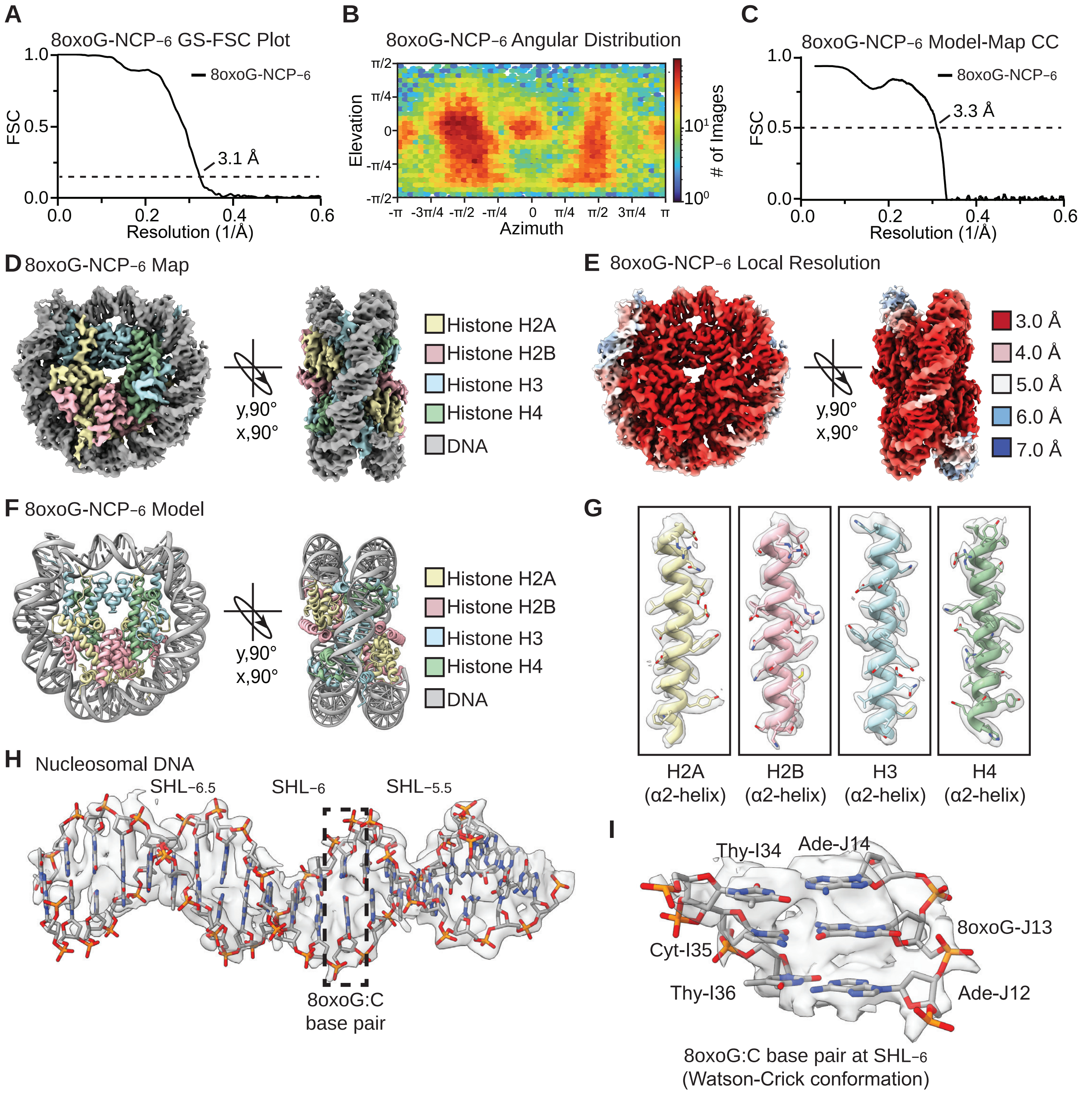


**Supplementary Figure 7: 8-oxoG-NCP$_{-6}$ map and model quality assessment.**
**A**, Gold-standard Fourier Shell Correlation (GS-FSC) for the 8-oxoG-NCP$_{-6}$. The dashed line corresponds to FSC-0.143. **B**, Angular distribution heatmap for the 8-oxoG-NCP$_{-6}$ cryo-EM map. **C**, Map-to-model FSC curves for the 8-oxoG-NCP$_{-6}$ model and 8-oxoG-NCP$_{-6}$ cryo-EM map. The dashed line corresponds to FSC-0.5. **D**, Final 3.1 Å cryo-EM map of 8-oxoG-NCP$_{-6}$ shown in two different orientations. **E**, Local resolution estimation for the 8-oxoG-NCP$_{-6}$ cryo-EM map shown in two different orientations. **F**, Final 8-oxoG-NCP$_{-6}$ model shown in two different views. **G**, Representative segmented density for histones H2A, H2B, H3, and H4 in the 8-oxoG-NCP$_{-6}$ cryo-EM map. **H**, Representative segmented density for the nucleosomal DNA (SHL$_{-5.5}$ to SHL$_{-6.5}$) in the 8-oxoG-NCP$_{-6}$ cryo-EM map. The black dashed box highlights the 8-oxoG:C base pair at SHL$_{-6}$. **I**, Representative segmented density for the Watson-Crick 8-oxoG:C base pair at SHL$_{-6}$ in the 8-oxoG-NCP$_{-6}$ cryo-EM map. Related to Figure 4.

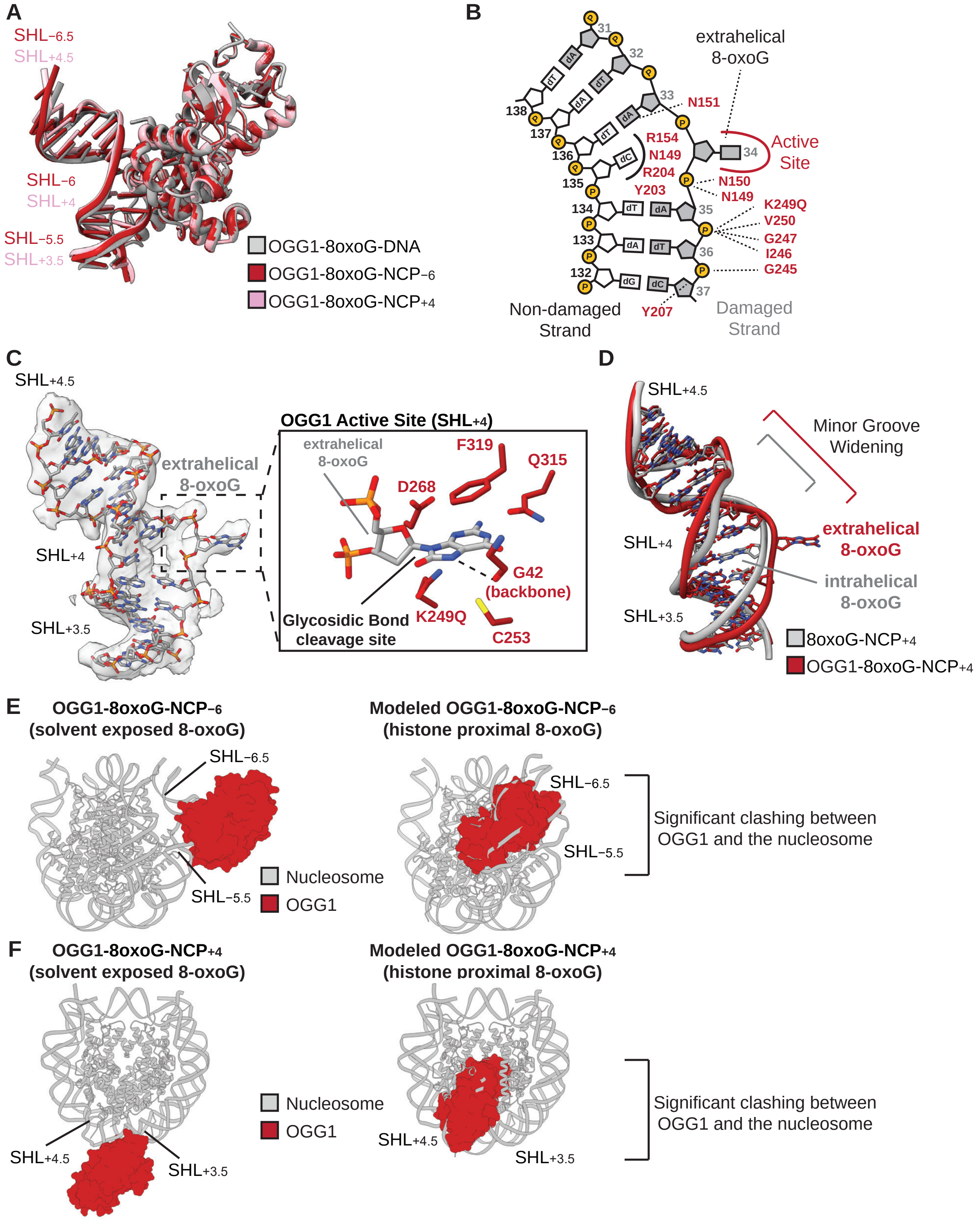


**Supplementary Figure 8: A conserved OGG1 8-oxoG recognition mechanism at $SHL_{+4}$.** **A,** Structural comparison of OGG1 and the nucleosomal DNA in the OGG1-8-oxoG-$NCP_{-6}$, OGG1-8-oxoG-$NCP_{+4}$, and OGG1-8-oxoG-DNA (1EBM) complexes. **B**, A diagram representing the interactions between OGG1 and the nucleosomal DNA in the OGG1-8-oxoG-$NCP_{+4}$ complex identified using PLIP92. **C**, Focused view of the nucleosomal DNA at $SHL_{+3.5}$ to $SHL_{+4.5}$ showing the extrahelical 8-oxoG at $SHL_{+4}$. The segmented density for the nucleosomal DNA in the OGG1-8-oxoG-$NCP_{+4}$ composite cryo-EM map is shown in transparent grey. An inset of the OGG1 active site is shown highlighting key amino acids important for 8-oxoG recognition and excision. **D**, Structural comparison of the nucleosomal DNA ($SHL_{+3.5}$ to $SHL_{+4.5}$) in the OGG1-8-oxoG-$NCP_{+4}$ complex and 8-oxoG-$NCP_{+4}$. **E**, OGG1-8-oxoG-$NCP_{-6}$ cryo-EM model (left) and a structural model of OGG1 bound to a histone proximal 8-oxoG at $SHL_{-6}$ (right), showing significant clashes between OGG1 and the nucleosome when 8-oxoG is adjacent to the histone octamer. **F**, OGG1-8-oxoG-$NCP_{+4}$ cryo-EM model (left) and a structural model of OGG1 bound to a histone proximal 8-oxoG at $SHL_{+4}$ (right), showing significant clashes between OGG1 and the nucleosome when 8-oxoG is adjacent to the histone octamer. Related to Figure 4.

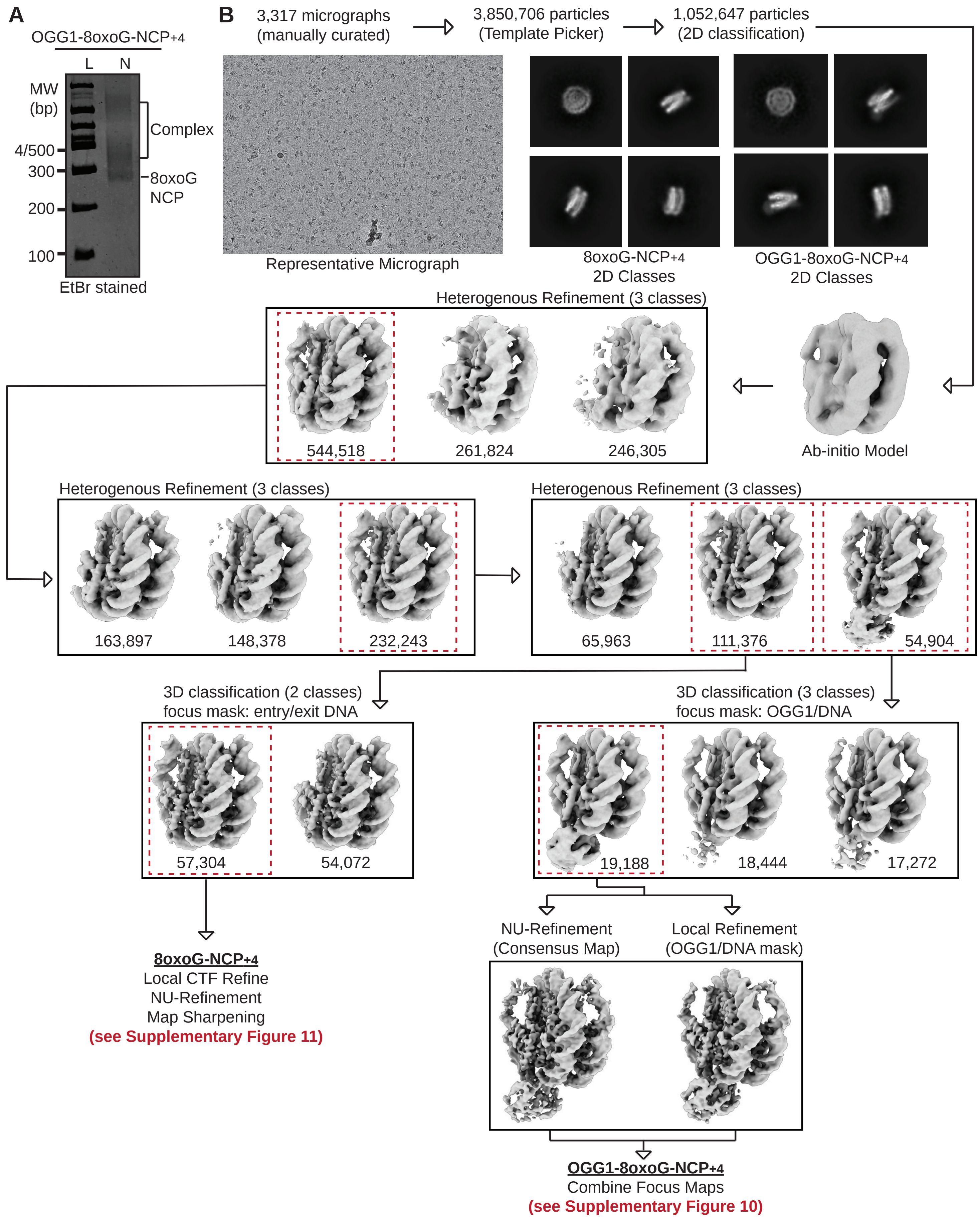


**Supplementary Figure 9: Single particle analysis of OGG1-8-oxoG-NCP$_{+4}$.**
**A,** Native PAGE gel of the OGG1-8-oxoG-NCP$_{+4}$ cryo-EM sample (S) and a 100 bp DNA ladder (L). The nucleosome complex was visualized with ethidium bromide staining. **B**, Flowchart of the data processing pipeline for the OGG1-8-oxoG-NCP$_{+4}$ cryo-EM dataset. A representative micrograph (n=3,317) and representative 2D classes from the OGG1-8-oxoG-NCP$_{+4}$ cryo-EM dataset are shown. All maps chosen for the downstream processing are labeled by a dotted red box. The final maps, models, and quality assessment for the OGG1-8-oxoG-NCP$_{+4}$ and 8-oxoG-NCP$_{+4}$ structures can be found in Supplementary Figure 10 and 11, respectively. Related to Figure 4.

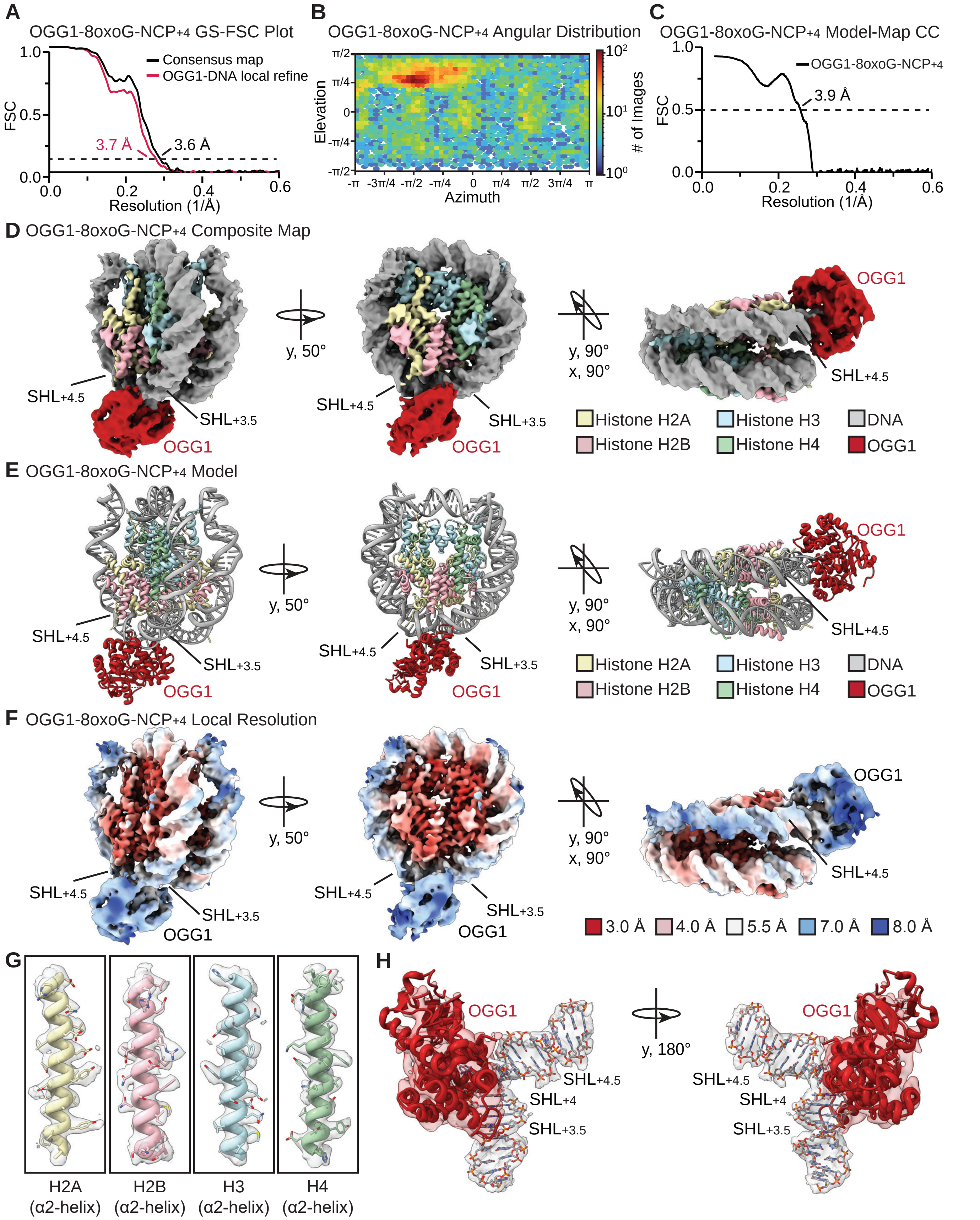


**Supplementary Figure 10: OGG1-8-oxoG-NCP$_{+4}$ map and model quality assessment.**
**A,** Gold-standard Fourier shell correlation (GS-FSC) curves for the OGG1-8-oxoG-NCP$_{+4}$ (consensus, black line) and OGG1-8-oxoG-NCP$_{+4}$ (OGG1/DNA focus, red line) cryo-EM maps. The dashed line corresponds to FSC-0.143. **B**, Angular distribution heatmap for the 8-oxoG-NCP$_{+4}$ cryo-EM maps. **C**, Map-to-model FSC curves for the OGG1-8-oxoG-NCP$_{+4}$ model and OGG1-8-oxoG-NCP$_{+4}$ composite cryo-EM map. **D**, Final 3.6 Å OGG1-8-oxoG-NCP$_{+4}$ composite cryo-EM map shown in three different orientations. **E**, Final OGG1-8-oxoG-NCP$_{+4}$ model shown in three different orientations. **F**, Local resolution estimation for the OGG1-8-oxoG-NCP$_{+4}$ composite cryo-EM map shown in three different orientations. **G**, Representative segmented density for histones H2A, H2B, H3, and H4 in the OGG1-8-oxoG-NCP$_{+4}$ composite cryo-EM map. **H**, Representative segmented density for OGG1 and the nucleosomal DNA (SHL$_{+3.5}$ to SHL$_{+4.5}$) in the OGG1-8-oxoG-NCP$_{+4}$ cryo-EM map. Related to Figure 4.

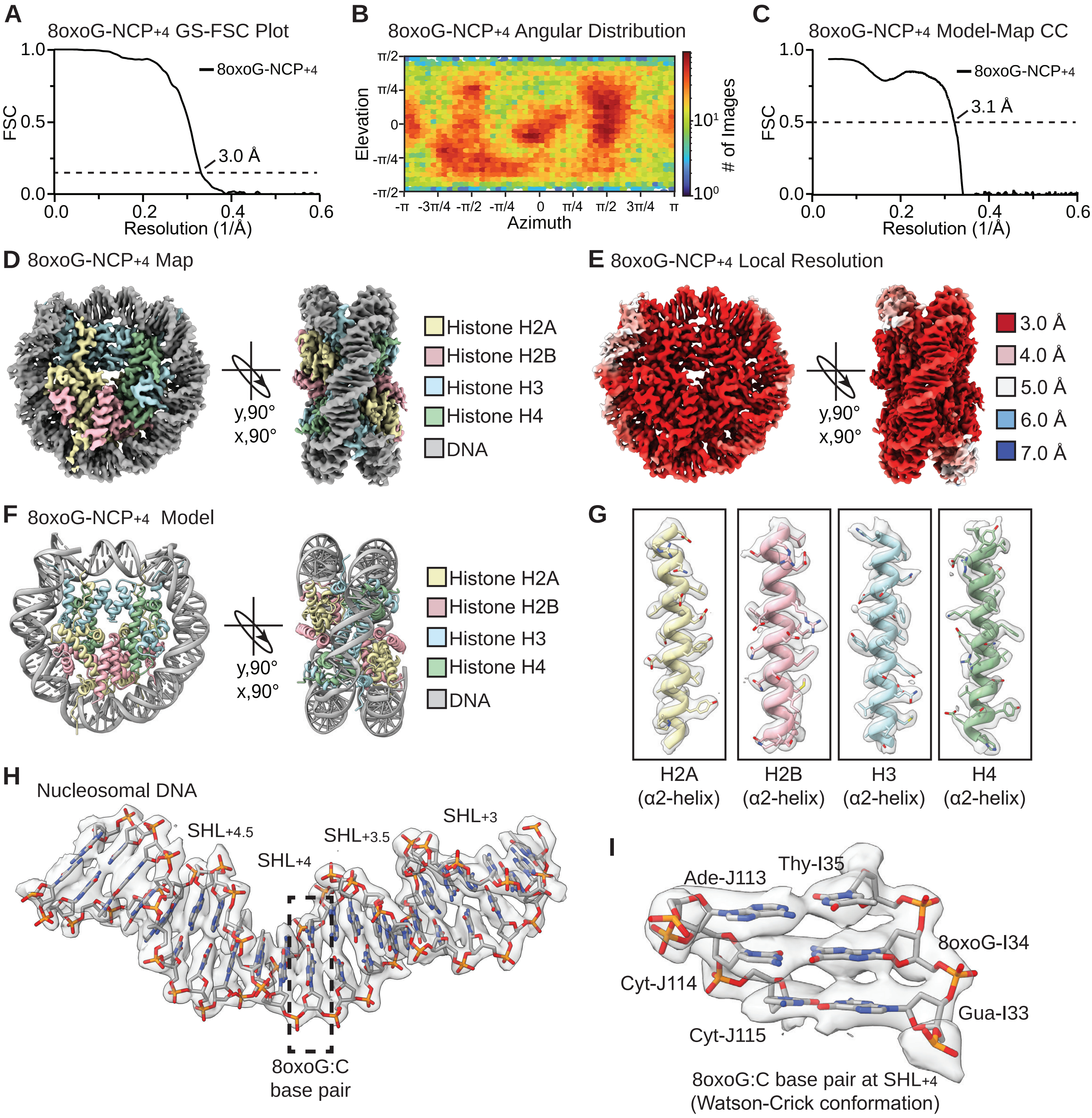


**Supplementary Figure 11: 8-oxoG-NCP+4 map and model quality assessment.**
**A,** Gold-standard Fourier Shell Correlation (GS-FSC) for the 8-oxoG-NCP$_{+4}$. The dashed line corresponds to FSC-0.143. **B**, Angular distribution heatmap for the 8-oxoG-NCP$_{+4}$ cryo-EM map. **C**, Map-to-model FSC curves for the 8-oxoG-NCP+4 model and 8-oxoG-NCP+4 cryo-EM map. The dashed line corresponds to FSC-0.5. **D**, Final 3.0 Å cryo-EM map of 8-oxoG-NCP$_{+4}$ shown in three different orientations. **E**, Local resolution estimation for the 8-oxoG-NCP$_{+4}$ cryo-EM map shown in two different orientations. **F**, Final 8-oxoG-NCP$_{+4}$ model shown in two different views. **G**, Representative segmented density for histones H2A, H2B, H3, and H4 in the 8-oxoG-NCP$_{+4}$ cryo-EM map. **H**, Representative segmented density for the nucleosomal DNA (SHL$_{+3}$ to SHL$_{+4.5}$) in the 8-oxoG-NCP$_{+4}$ cryo-EM map. The black dashed box highlights the 8-oxoG:C base pair at SHL$_{+4}$. **I**, Representative segmented density for the Watson-Crick 8-oxoG:C base pair at SHL$_{+4}$ in the 8-oxoG-NCP$_{+4}$ cryo-EM map. Related to Figure 4.

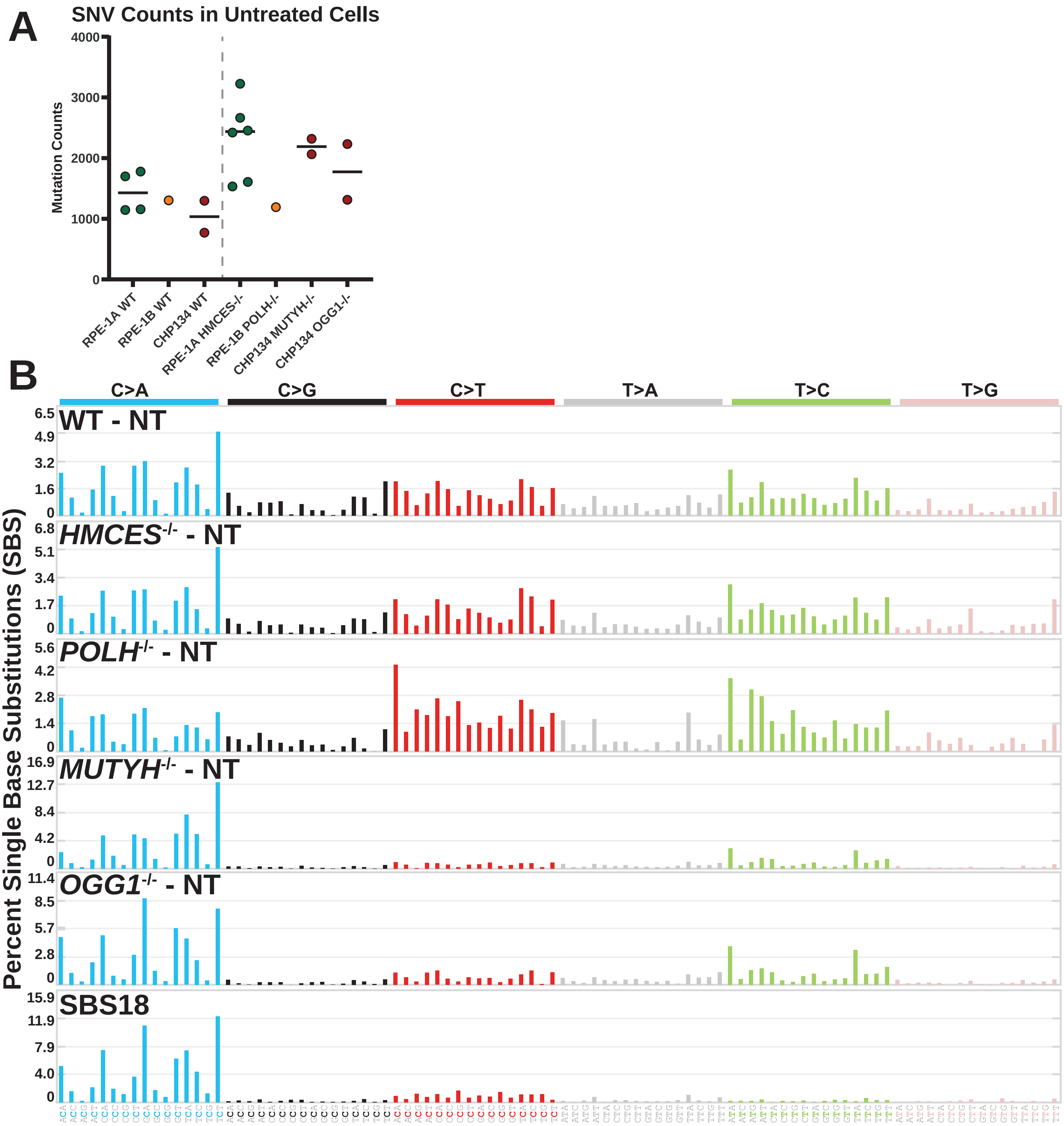


**Supplementary Figure 12: Spontaneous SNV spectra in untreated human cells with 8-oxoG associated gene knockouts.**

**A,** Number of SNVs per genome in untreated human cells. Circles indicate biologically independent genomes sequenced (n=4, 1, 2, 6, 1, 2, and 2 for RPE-1A WT, RPE-1B WT, CHP134 WT, RPE-1A *HMCES*$^{-/-}$, RPE-1B *POLH*$^{-/-}$, CHP134 *MUTYH*$^{-/-}$, and CHP134 *OGG1*$^{-/-}$, respectively). Horizontal bars are median values. **B**, Spectra plots of spontaneous SNVs in human WT, *HMCES*$^{-/-}$, *POLH*$^{-/-}$, *MUTYH*$^{-/-}$, and *OGG1*$^{-/-}$ cells along with the mutation spectra from SBS18. Related to Figure 5.

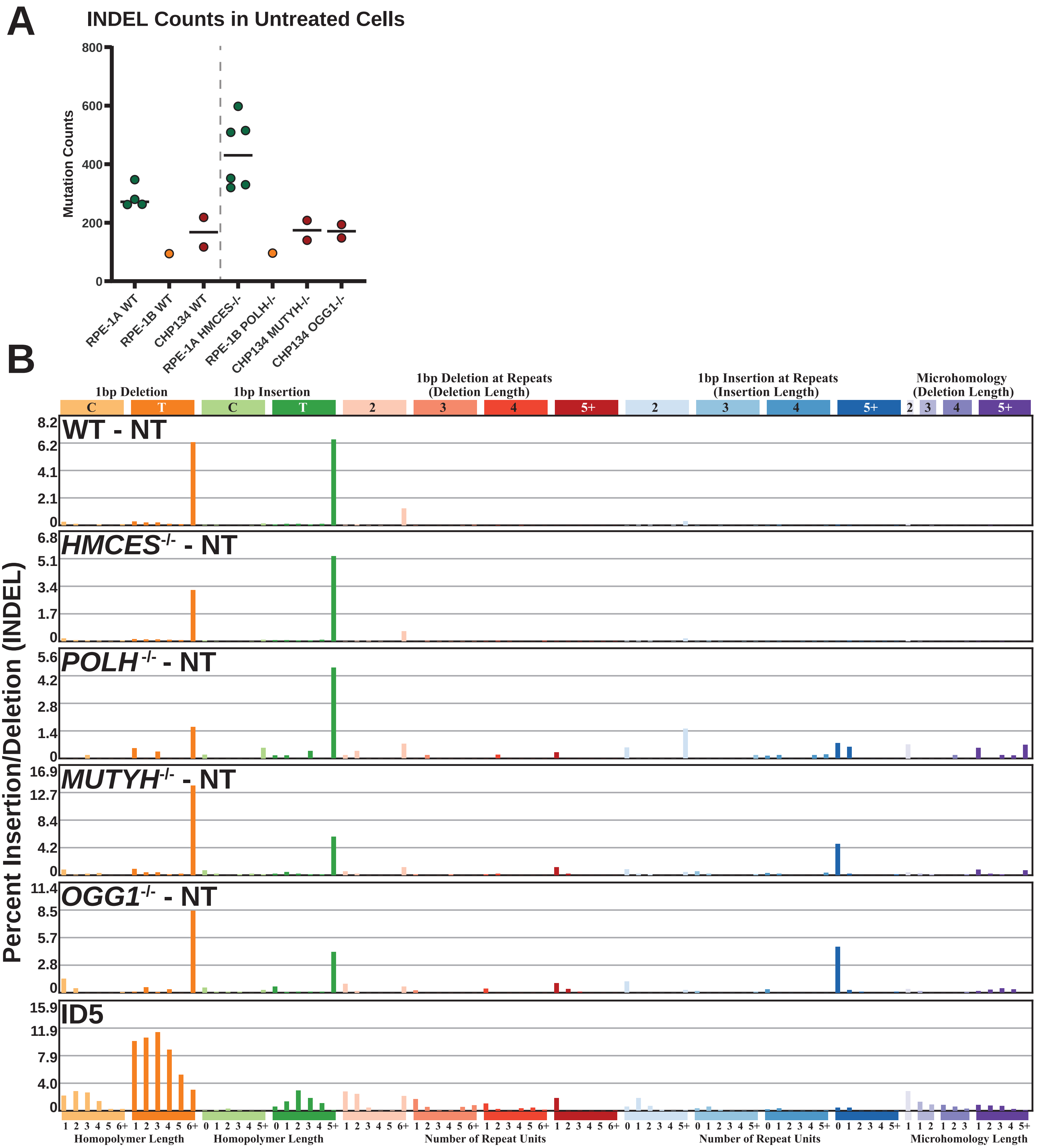


**Supplementary Figure 13: Spontaneous INDEL mutation spectra in untreated human cells with 8-oxoG associated gene knockouts.**

**A,** Number of INDELs per genome in untreated human cells. Circles indicate independent genomes sequenced. Horizontal bars are median values. **B**, Spectra plots of spontaneous INDELs in human WT, *HMCES*$^{-/-}$, *POLH*$^{-/-}$, *MUTYH*$^{-/-}$, and *OGG1*$^{-/-}$ cells along with the mutation spectra from ID5. Related to Figure 5.

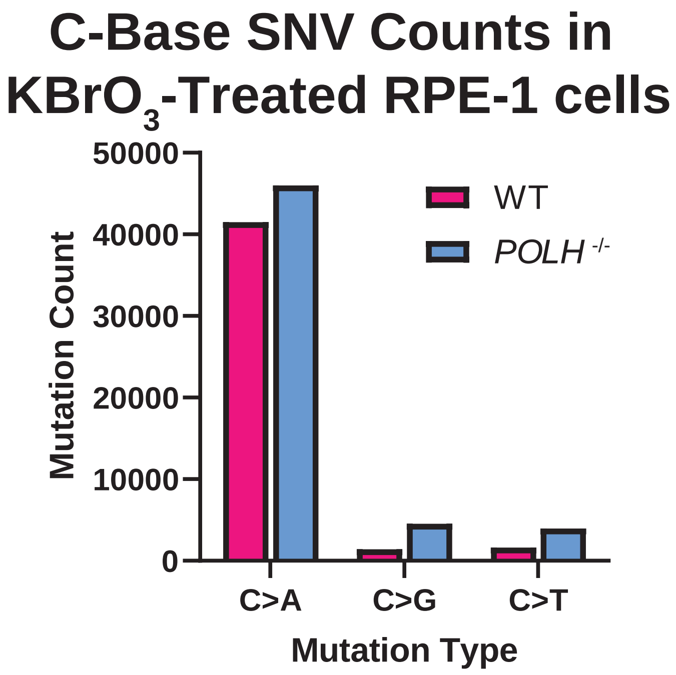


**Supplementary Figure 14: C-based SNV counts of $KBrO_3$ treated RPE-1 cells is increased in *POLH*$^{-/-}$ compared to WT.**
C>A, C>G, and C>T mutations were elevated by 0.11-, 2.14-, and 1.43-fold, respectively, in *POLH*$^{-/-}$ cells. The proportions of substitutions in the *POLH*$^{-/-}$ cells are statistically different compared to WT cells using a 2-way ANOVA (p=0.0345). Data represents mutation counts from one sample from each genotype obtained from[50].

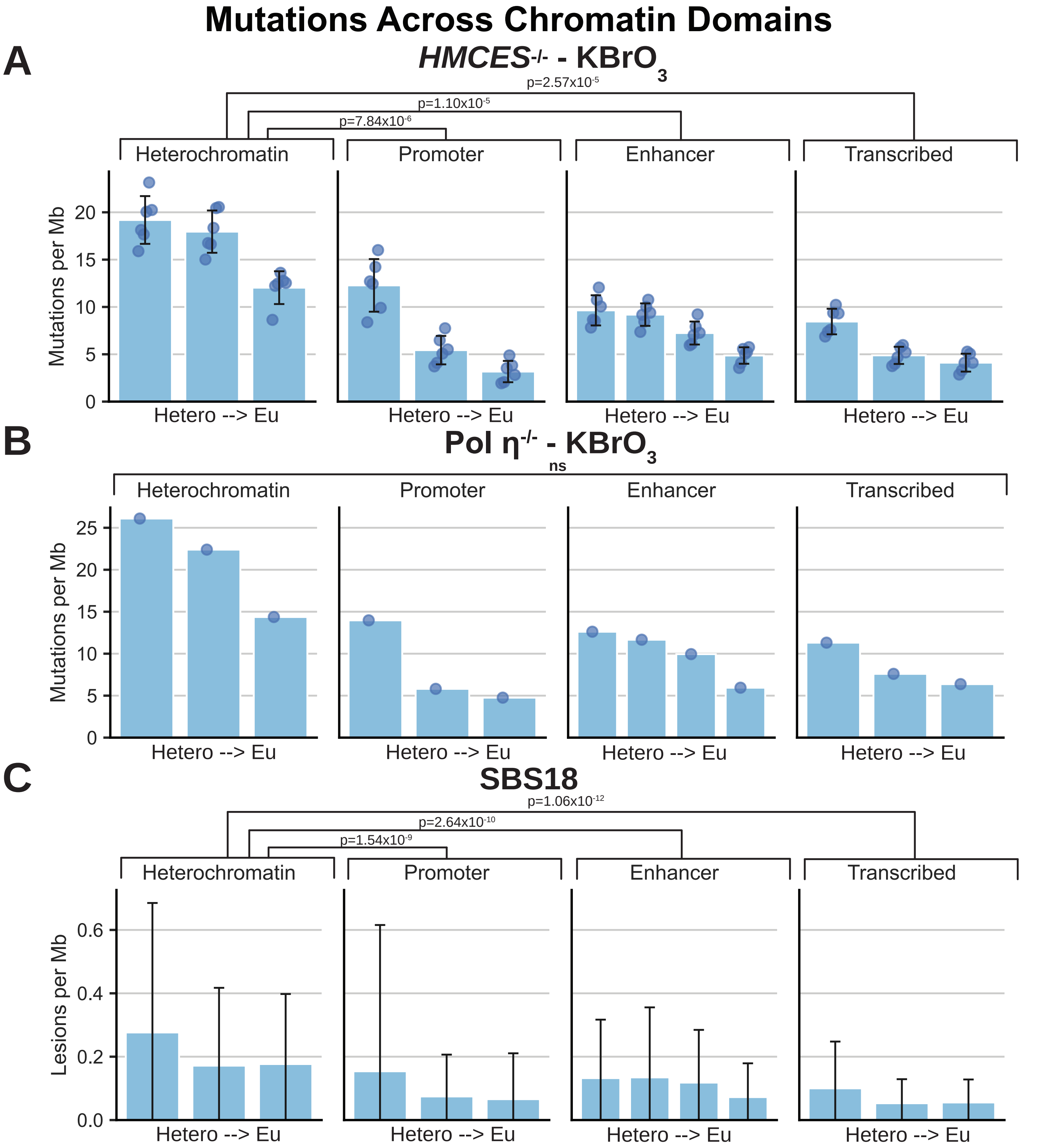


**Supplementary Figure 15: The impact of chromatin state on 8-oxoG mutagenesis in human cells.**
Four binned broad chromatin regions are dictated by the ChromHMM map and within groups are sorted from left to right in becoming increasingly more euchromatic. Normalized mapped mutations were graphed for (**A**) *HMCES*$^{-/-}$ RPE-1 cells (n=4), (**B**) *POLH*$^{-/-}$ RPE-1 cells (n=1) and (**C**) mutations from PCAWG tumors that have a 70% or greater attribution to SBS18 (n=99). Bars represent mean values, circles represent biologically independent sequenced genomes, and error bars represent standard deviation. Mutation rates in heterochromatin compared to promotor regions, enhancer regions, and transcribed regions were significantly different in (**A**) *HMCES*$^{-/-}$ and (**C**) SBS18. Precise p-values are indicated in the figure and were determined as Bonferroni-corrected two-sided paired t-test. ns indicates not significant. Related to Figure 6.

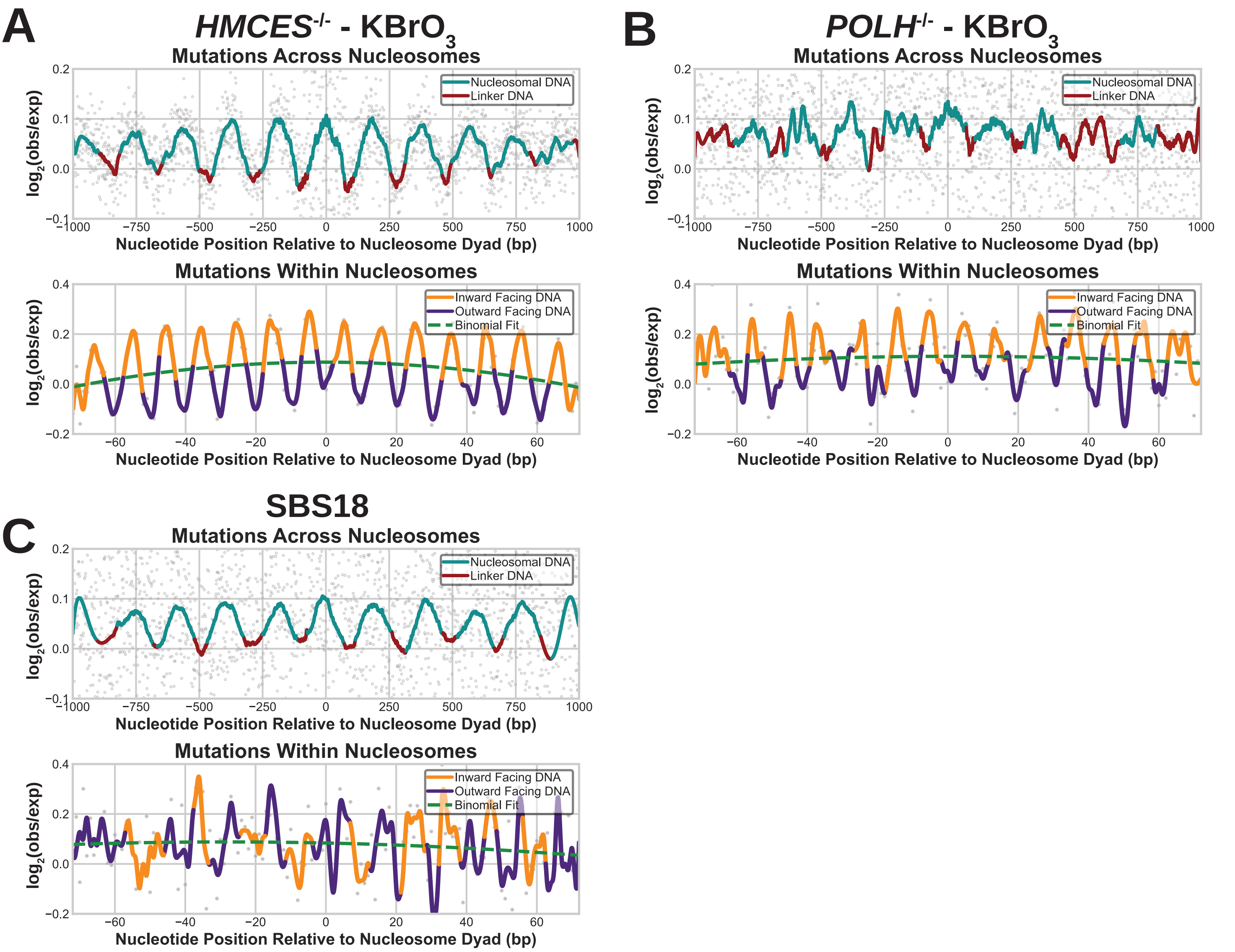


**Supplementary Figure 16: The impact of chromatin structure on 8-oxoG mutagenesis in human cells.**
The top graphs represent translational periodicity of $log_2$(observed/expected) of mutations between nucleosomes. Nucleosome bound DNA is represented in blue and linker DNA is represented in red. The bottom graphs represent the rotational periodicity of the $log_2$(observed/expected) of mutations within the nucleosome where DNA that is inward histone facing DNA relative to the nucleosome is displayed in gold while outward solvent facing DNA relative to the nucleosome is displayed in purple. A binomial fit of the data is overlayed in a dashed green line. In both figures, $log_2$(observed/expected) values for each position are displayed as gray circles. This analysis was completed for (**A**) *HMCES$^{-/-}$* RPE-1 cells, (**B**) *POLH$^{-/-}$* RPE-1 cells and (**C**) mutations from PCAWG that have a 70% or greater attribution to SBS18. Related to Figure 3.

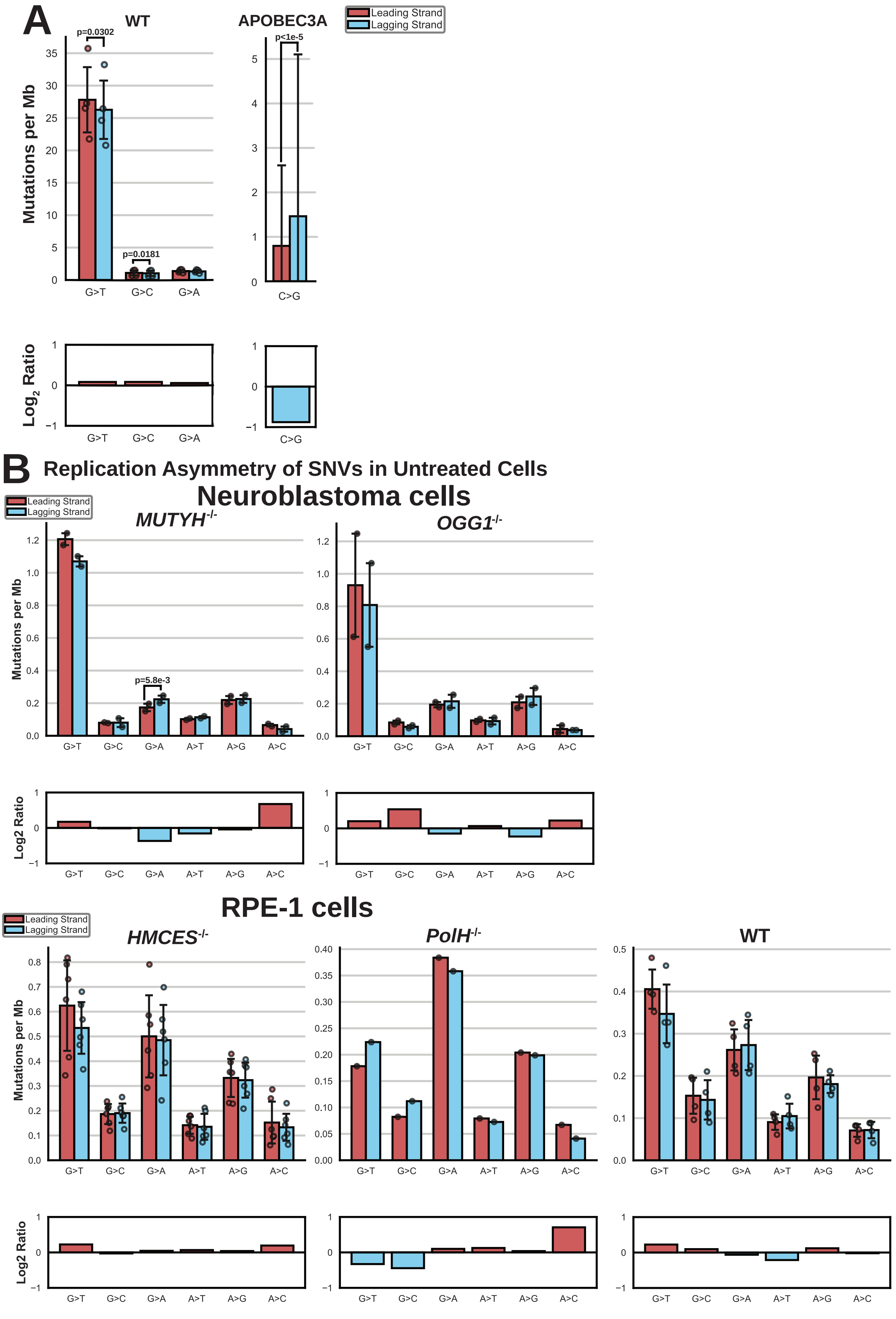


**Supplementary Figure 17: Replication asymmetry of $KBrO_3$ mutagenesis, APOBEC mutagenesis, and DNA repair deficiencies.**

**A**, Mean densities of G>T, G>C, G>A, and C>G mutations on the leading strand template (red bars) and lagging strand template (blue bars). G>T, G>C, and G>A substitutions occur in $KBrO_3$-treated WT cells and C>G substitutions were obtained from whole genome sequenced breast cancers[93], containing the APOBEC mutation signature. **B**, Mean densities of G or A substitutions on the leading strand (red bars) and lagging strand (blue bars) in *OGG1*$^{-/-}$ and *MUTYH*$^{-/-}$ neuroblastoma cells or WT, *HMCES*$^{-/-}$, and *POLH*$^{-/-}$ RPE-1 cells. The bar graph below each plot represents the $\log_2$(red/blue) value of the for the event per Mb bars. circles represent biologically independent sequenced genomes (WT: n=4, APOBEC: n=270, *MUTYH*$^{-/-}$: n=2, *OGG1*$^{-/-}$: n=2, *HMCES*$^{-/-}$: n=6, *POLH*$^{-/-}$: n=1), and the error bars represent standard deviation. Significant differences between strands indicated with p-values determined by two-sided paired t-test.

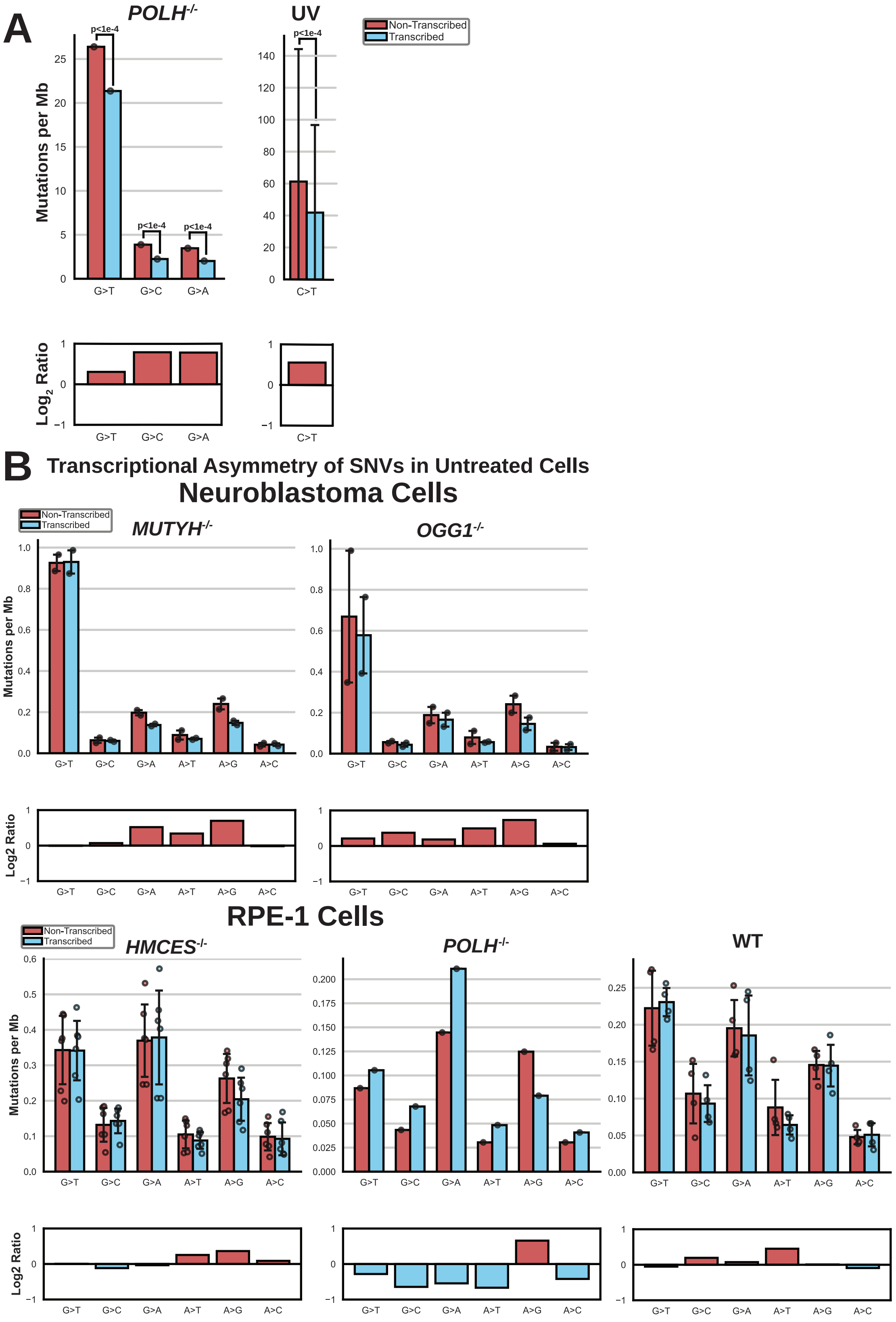


**Supplementary Figure 18: Transcriptional asymmetry of $KBrO_3$ mutagenesis, UV mutagenesis, and DNA repair deficiencies.**

**A**, Mean densities of G>T, G>C, G>A, or C>T mutations on the non-transcribed (red bars) and transcribed (blue bars) strands. G>T, G>C, and G>A substitutions are from $KBrO_3$-treated *POLH*$^{-/-}$ cells and dipyrimidine C>T substitutions from sequenced human melanomas[91] **B**, Mean densities of G or A substitutions on the non-transcribed (red bars) and transcribed (blue bars) strand in *OGG1*$^{-/-}$ and *MUTYH*$^{-/-}$ neuroblastoma cells or WT, *HMCES*$^{-/-}$, and *POLH*$^{-/-}$ RPE-1 cells. The bar graph below each plot represents the $\log_2$(red/blue) value of the for the event per MB bars. circles represent biologically independent sequenced genomes (WT: n=4, Melanoma: n=70, *MUTYH*$^{-/-}$: n=2, *OGG1*$^{-/-}$: n=2, *HMCES*$^{-/-}$: n=6, *POLH*$^{-/-}$: n=1), and the error bars represent standard deviation. Significant differences between strands in WT, melanoma, *MUTYH*$^{-/-}$, *OGG1*$^{-/-}$, and *HMCES*$^{-/-}$ samples are indicated in with p-values determined by two-sided paired t-test. p-values for G base mutation types in the *POLH*$^{-/-}$ sample were calculated by two-sided Chi-square against a null hypothesis of equal occurrence on both DNA strands.
