# Supplementary material for: Contributing factors to the oxidation-induced mutational landscape in human cells
Source: Nat Commun. 2024 Dec 23;15:10722. doi: 10.1038/s41467-024-55497-z (PMC11666792; doi:10.1038/s41467-024-55497-z)
Supplement: Supplementary file 2 — Description of Additional Supplementary Information [file 41467_2024_55497_MOESM2_ESM.docx]

**Description of Additional Supplementary Files**

File Name: Supplementary Data 1

Description: List of mutations from whole genome sequencing of WT, HMCESdeficient, and Pol η-deficient RPE-1 cells
