# Supplementary material for: Contributing factors to the oxidation-induced mutational landscape in human cells
Source: Nat Commun. 2024 Dec 23;15:10722. doi: 10.1038/s41467-024-55497-z (PMC11666792; doi:10.1038/s41467-024-55497-z)
Supplement: Supplementary file 4 — Reporting Summary [file 41467_2024_55497_MOESM4_ESM.pdf]

nature portfolio

Corresponding author(s): Steven A. Roberts, Bret Freudenthal, Kavi Mehta, David Cortez

Last updated by author(s): Dec. 4 2024

Reporting Summary

Nature Portfolio wishes to improve the reproducibility of the work that we publish. This form provides structure for consistency and transparency in reporting. For further information on Nature Portfolio policies, see our Editorial Policies and the Editorial Policy Checklist.

Statistics

For all statistical analyses, confirm that the following items are present in the figure legend, table legend, main text, or Methods section.

n/a	Confirmed	
[ ]	[x]	The exact sample size (n) for each experimental group/condition, given as a discrete number and unit of measurement
[ ]	[x]	A statement on whether measurements were taken from distinct samples or whether the same sample was measured repeatedly
[ ]	[x]	The statistical test(s) used AND whether they are one- or two-sided<br>*Only common tests should be described solely by name; describe more complex techniques in the Methods section.*
[x]	[ ]	A description of all covariates tested
[ ]	[x]	A description of any assumptions or corrections, such as tests of normality and adjustment for multiple comparisons
[ ]	[x]	A full description of the statistical parameters including central tendency (e.g. means) or other basic estimates (e.g. regression coefficient) AND variation (e.g. standard deviation) or associated estimates of uncertainty (e.g. confidence intervals)
[ ]	[x]	For null hypothesis testing, the test statistic (e.g. F, t, r) with confidence intervals, effect sizes, degrees of freedom and P value noted<br>*Give P values as exact values whenever suitable.*
[x]	[ ]	For Bayesian analysis, information on the choice of priors and Markov chain Monte Carlo settings
[x]	[ ]	For hierarchical and complex designs, identification of the appropriate level for tests and full reporting of outcomes
[ ]	[x]	Estimates of effect sizes (e.g. Cohen's d, Pearson's r), indicating how they were calculated

Our web collection on statistics for biologists contains articles on many of the points above.

Software and code

Policy information about availability of computer code

Data collection	All Cryo-EM data collection was performed using SerialEM at the The University of Chicago Advanced Electron Microscopy Core Facility (RRID:SCR_019198).
Data analysis	All cryo-EM data processing and analysis was performed in cryoSPARC v3.3 and/or v4. All initial rigid-body docking, model building and refinements were performed UCSF Chimera, UCSF ChimeraX v1.4, PHENIX v1.19.2-4158, and COOT v0.9.5. Model validation was performed using MolProbity v4.02b-467. Analysis of the OGG1 nucleosome binding interface was performed with PLIP v2.3. All whole genome sequencing data was analyzed using BWA v0.7.17, samtools v1.13 using htslib v1.13+ds, Strelka2 (v2.9.10), Manta (v1.6.0), VarScan2 (v2.3), Somatic Sniper (v1.0.5.0), SigProfilerExtractor (v1.1.23), MutationalPatterns.R, vcf2maf (v1.6.21), VEP (v102), and AsymTools2. 8-oxoG lesion mapping data was also aligned to the hg19 human reference genome using BWA v0.7.17.

For manuscripts utilizing custom algorithms or software that are central to the research but not yet described in published literature, software must be made available to editors and reviewers. We strongly encourage code deposition in a community repository (e.g. GitHub). See the Nature Portfolio guidelines for submitting code & software for further information.

Data

Policy information about availability of data

All manuscripts must include a data availability statement. This statement should provide the following information, where applicable:

- Accession codes, unique identifiers, or web links for publicly available datasets
- A description of any restrictions on data availability
- For clinical datasets or third party data, please ensure that the statement adheres to our policy

The next generation sequencing data generated in this study for untreated and KBrO3-treated hTERT-RPE-1 p53-/- and hTERT-RPE-1 p53-/- HMCES-/- cells have been deposited as FASTQ files at the NCBI SRA database under accession code PRJNA1100509. Full mutation lists used for analysis are provided in Supplementary Data 1. hTERT-RPE-1 POLH-/- VCF files used in this study are available from50 available on Mendeley Data server (https://doi.org/10.17632/jkjkpvgxyd.1). FASTQ files for KBrO3-treated hTERT-RPE-1 POLH-/- cells used I this study are available in NCBI SRA database under accession code PRJNA940340. MUTYH-/- and OGG1-/- VCF files can be obtained from the supplementary dataset S01 from8. CLAPS-seq 8-oxoG lesion mapping data from39 are available from the Gene Expression Omnibus (GEO) (https://www.ncbi.nlm.nih.gov/geo/) under accession code GSE181312. Publicly available lists of tumor mutations were obtained from the International Cancer Genome Consortium (ICGC) from consensus_snv_indel/final_consensus_passonly.snv_mnv_indel.icgc.public.maf.gz and simple_somatic_mutation.open.BRCA-EU.tsv.gz. Corresponding tumor mutation lists can be downloaded from ICGC using the linked download instructions. The final cryo-EM maps are available from the Electron Microscopy Data Bank under accession numbers EMD-43600 for OGG1-8-oxoG-NCP−6 (composite), EMD-43597 for OGG1-8-oxoG-NCP−6 (consensus), EMD-43598 for OGG1-8-oxoG-NCP−6 (NCP local refine), EMD-43599 for OGG1-8-oxoG-NCP−6 (OGG1/DNA local refine), EMD-43601 for OGG1-8-oxoG-NCP+4 (composite), EMD-43602 for OGG1-8-oxoG-NCP+4 (consensus), and EMD-43603 for OGG1-8-oxoG-NCP+4 (OGG1/DNA local refine). The model coordinates for each structure are available from the Protein Data Bank (PDB) under accession numbers 8VWS for 8-oxoG-NCP−6, 8VWT for OGG1-8-oxoG-NCP−6, 8VWU for 8-oxoG-NCP+4, 8VWV for OGG1-8-oxoG-NCP+4. All data is publicly available and accessible without restriction. Values underlying all graphs in figures are provided in the Source Data file.

Research involving human participants, their data, or biological material

Policy information about studies with human participants or human data. See also policy information about sex, gender (identity/presentation), and sexual orientation and race, ethnicity and racism.

Reporting on sex and gender	All experiments in this work was completed either with commercially available immortalized human cell lines or publicly available sequencing data/mutation lists from commerically available cell lines or tumor samples. As such, this work is not human subjects research and therefore no reporting of sex or gender is involved.
Reporting on race, ethnicity, or other socially relevant groupings	All experiments in this work was completed either with commercially available immortalized human cell lines or publicly available sequencing data/mutation lists from commerically available cell lines or tumor samples. As such, this work is not human subjects research and therefore no reporting of rare, ethnicity or social group is involved.
Population characteristics	All experiments in this work was completed either with commercially available immortalized human cell lines or publicly available sequencing data/mutation lists from commerically available cell lines or tumor samples. As such, this work is not human subjects research and no population characteristics are involved.
Recruitment	All experiments in this work was completed either with commercially available immortalized human cell lines or publicly available sequencing data/mutation lists from commerically available cell lines or tumor samples. As such, this work is not human subjects research and there was no participate recruitment.
Ethics oversight	All experiments in this work was completed either with commercially available immortalized human cell lines or publicly available sequencing data/mutation lists from commerically available cell lines or tumor samples. As such, this work is not human subjects research and does not require an IRB protocol.

Note that full information on the approval of the study protocol must also be provided in the manuscript.

Field-specific reporting

Please select the one below that is the best fit for your research. If you are not sure, read the appropriate sections before making your selection.

[x] Life sciences [ ] Behavioural & social sciences [ ] Ecological, evolutionary & environmental sciences

For a reference copy of the document with all sections, see nature.com/documents/nr-reporting-summary-flat.pdf

Life sciences study design

All studies must disclose on these points even when the disclosure is negative.

Sample size	For WT and HMCES knockout cells sequenced as part of this study, at least 4 independent cell isolates were sequenced for each condition to allow a sufficient number of replicates to statistically evaluate differences in mutation load and spectrum by T-test or non-parametric Mann-Whitney. The number of total mutations from this sample size was also sufficient for mutation topology analyses that assessed regional differences in mutation density on the aggregate sequencing data. Sample size was not predetermined for the cryo-EM experiments. The sample size was determined by the number of particles that could be reasonably obtained during 1 day of data collection. This yielded structures with a resolution of ~4Å or better, which was sufficient for model building into the cryo-EM maps.

Data exclusions	No Data were excluded from the cell biology and sequencing analyses. Some individual micrographs and/or particles from the cryo-EM datasets were excluded during data processing. The cryo-EM datasets were subjected to manual micrograph curation in order to exclude poor micrgraphs resulting from significant ice contamination, poor CTF fit, and/or elevated motion during data collection. In addition, all cryo-EM datasets were subjected to extensive 2D and 3D classification during data processing to remove low quality particles and/or classes of particles. These practices are standard within the cryo-EM field and enable the generation of interpretable high-resolution cryo-EM maps. See Sigworth, Microscopy (Oxf), 2015. PMCID: PMC4749045.
Replication	Sequencing was conducted on at least 4 independent WT and HMCES knockout cell isolates. All replicates produced similar numbers and spectra of mutations indicating that the experimental process was highly reproducible.
Randomization	Mutation counts determined from sequencing of cell isolates from different genotypes were used in this manuscript. Since these numbers are objectively obtained directly from the sequencing data, no randomization was conducted or is required.
Blinding	Mutation counts determined from sequencing of cell isolates from different genotypes were used in this manuscript. Since these numbers are objectively obtained directly from the sequencing data, no blinding was conducted or is required.

Reporting for specific materials, systems and methods

We require information from authors about some types of materials, experimental systems and methods used in many studies. Here, indicate whether each material, system or method listed is relevant to your study. If you are not sure if a list item applies to your research, read the appropriate section before selecting a response.

Materials & experimental systems

n/a	Involved in the study
☒	☐ Antibodies
☐	☒ Eukaryotic cell lines
☒	☐ Palaeontology and archaeology
☒	☐ Animals and other organisms
☒	☐ Clinical data
☒	☐ Dual use research of concern
☒	☐ Plants

Methods

n/a	Involved in the study
☒	☐ ChIP-seq
☒	☐ Flow cytometry
☒	☐ MRI-based neuroimaging

Eukaryotic cell lines

Policy information about cell lines and Sex and Gender in Research

Cell line source(s)	Human Retinal Epithelial Cells were obtained from Dr. Dan Durocher (Lunenfeld-Tanenbaum Research Institute).
Authentication	RPE-1 cells used in this manuscript displayed the expected cell morphology and growth characteristics for the cell line.
Mycoplasma contamination	RPE-1 cells were mycoplasma tested and confirmed negative.
Commonly misidentified lines (See ICLAC register)	no commonly misidentified lines were grown for this manuscript.

Plants

Seed stocks	No Plants were used in this study.
Novel plant genotypes	No Plants were used in this study.
Authentication	No Plants were used in this study.
